# Leaf plasticity across wet and dry seasons in *Croton blanchetianus* (Euphorbiaceae) at a tropical dry forest

**DOI:** 10.1038/s41598-022-04958-w

**Published:** 2022-01-19

**Authors:** Keila Rêgo Mendes, Willian Batista-Silva, Jaqueline Dias-Pereira, Marcos P. S. Pereira, Eliane V. Souza, José E. Serrão, João A. A. Granja, Eugênia C. Pereira, David J. Gallacher, Pedro R. Mutti, Duany T. C. da Silva, Rogério S. de Souza Júnior, Gabriel B. Costa, Bergson G. Bezerra, Cláudio M. Santos e Silva, Marcelo F. Pompelli

**Affiliations:** 1 Department of Atmospheric and Climate Sciences, Federal University of Rio Grande Do Norte, Av. Senador Salgado Filho, 3000, Lagoa Nova, Natal, 59078-970 Brazil; 2 Plant Physiology Graduate Program, Department of Plant Biology, Federal University of Viçosa, Viçosa, MG Brazil; 3 Plant Anatomy Laboratory, Federal University of Viçosa, Rio Paranaíba, MG Brazil; 4 State Secretariat of Environment and Water Resources of Alagoas, Maceió, AL Brazil; 5 Department of Biology, Federal University of Viçosa, Viçosa, MG Brazil; 6 School Aggeu Magalhães, Serra Talhada, PE Brazil; 7 Department of Geographical Sciences, Federal University of Pernambuco, Recife, PE Brazil; 8 Plant Breeding Institute, The University of Sydney, Narrabri, NSW Australia; 9 Institute of Engineering and Geosciences, Federal University of West Pará, Rua Vera Paz s/n, Salé, Santarém, 68035-110 Brazil; 10 Federal University of Pernambuco, Recife, PE 50670901 Brazil; 11 Institute of Biodiversity and Forests, Federal University of West Pará, Rua Vera Paz s/n, Salé, Santarém, 68035-110 Brazil; 12 Grupo Regional de Investigación Participativa de los Pequeños Productores de La Costa Atlantica. Universidad de Córdoba, Carrera 6 No. 77- 305, Montería, Córdoba, Colombia

**Keywords:** Plant sciences, Ecology, Environmental sciences

## Abstract

Plant species of the Brazilian Caatinga experience seasonal wet and dry extremes, requiring seasonally different leaf characteristics for optimizing water availability. We investigated if *Croton blanchetianus* Baill exhibits leaf morphoanatomical traits across seasons and positioning in sunlight/natural shade. Leaves of ten 1-3 m tall plants in full sunlight and ten in natural shade were assessed in May, July (wet season), October and December (dry season) 2015 for gas exchange, leaf size, lamina and midrib cross sections (14 parameters), and chloroplast structure (5 parameters). Net photosynthesis was greater during the wet season (21.6 µm^−2^ s^−1^) compared to the dry season (5.8 µm^−2^ s^−1^) and was strongly correlated with almost all measured parameters (*p* < 0.01). Shaded leaves in the wet season had higher specific leaf area (19.9 m^2^ kg^−1^ in full-sun and 23.1 m^2^ kg^−1^ in shade), but in the dry season they did not differ from those in full sun (7.5 m^2^ kg^−1^ and 7.2 m^2^ kg^−1^). In the wet season, the expansion of the adaxial epidermis and mesophyll lead to larger and thicker photosynthetic area of leaves. Furthermore, chloroplast thickness, length and area were also significantly larger in full sunlight (2.1 μm, 5.1 μm, 15.2 μm^2^; respectively) and shaded plants (2.0 μm, 5.2 μm, 14.8 μm^2^; respectively) during wetter months. *Croton blanchetianus* exhibits seasonal plasticity in leaf structure, presumably to optimize water use efficiency during seasons of water abundance and deficit. These results suggest that the species is adaptable to the increased drought stress projected by climate change scenarios.

## Introduction

Morphological plasticity in response to prevailing conditions is a common feature in plants. For example, many woody species respond to herbivory by adapting their macrostructure and the frequency or size of thorns^1^. Biomes with distinct wet and dry seasons provide conditions under which leaf morphological plasticity would be advantageous, since the optimal leaf structure for plant growth varies with season^2–4^. Seasonal variations in water availability have been shown to affect new leaf length and pubescence in *Encelia farino*^5^, leaf rolling of *Phlomis fruticosa*^6^, and both internal and external leaf structure of several tree species^7^. However, information on leaf trait plasticity across habitats and seasons is limited^8–10^. Several studies showed that leaf nitrogen content, leaf lifespan, photosynthetic rate, and specific leaf area are associated with major biomes, functional groups, and species^3,11–13^ but information on interseasonal plasticity within individual perennial plants is limited.

Plant tolerance to periodic water deficit is expected to be of increasing importance as climate change intensifies inter- and intra-annual rainfall extreme^14–18^ . Water deficit is the primary cause of plant stress in arid and semiarid regions^2,3,18–21^. In such habitats, a species’ survival is dependent on its ability to adjust growth and development to utilize water when abundant, but be resilient to water stress when it is not^11^.

The Caatinga region of Brazil is characterized as semiarid tropical dry forest, with much of it’s low annual rainfall (300–1000 mm) occurring during intense summer storms^3^. Such habitats are structurally diverse, with plant functional groups exhibiting traits that are adapted to seasonal aridity^13,22^. Ecological research in the Caatinga has seen a recent focus on physiological mechanisms for adapting to seasonal water stress^2–4^ but they remain poorly understood. Light intensity also varies temporally with season and spatially with shading. Reduced light intensity is associated with an increased proportion of shoots to roots, reduced auto-shading, reduced palisade mesophyll and thus thinner leaves^23,24^, increased specific leaf area^4,25^, reduced stomatal density^24,26^, and increased leaf longevity^27,28^.

Any water deficit limits plant growth rate, impacting recruitment of natural populations and performance of crops^29,30^. Leaf anatomy of field-grown avocado cultivars (*Persea americana*) was strongly affected by water deficit, causing a 20% and 24% decline in absolute and specific leaf area, respectively, and a substantial reduction of palisade mesophyll^31^. However, potted olive trees (*Olea europaea*) showed a 17 and 22% increase in palisade and spongy mesophyll under water stress^32^. Thicker mesophyll could contain more sites of CO_2_ fixation^33^, whereas more spongy mesophyll could facilitate the diffusion of CO_2_ to the carboxylation sites. Several studies have indicated that leaves exposed to water stress have leaf midrib traits related to decreased leaf hydraulic conductance, such as xylem embolism^34–37^.

In the present study we investigated the morphoanatomical and ecophysiological mechanisms involved in drought tolerance of *Croton blanchetianus* Baill (Euphorbiaceae) plants throughout different seasons of the year, and which developed under both full-sun and natural shade conditions (i.e., May and July, here referred as wet season and October and December, here referred as dry season). Besides that, we have selected two types of leaves: those harvested under natural condition within irradiance around 70% of solar transmittance and lastly, plants under full sunlight environment. This Caatinga endemic species, *C*. *blanchetianus*, is a preferred livestock feed among indigenous graziers, and has a relatively high protein content^38^. It is an early colonizer after habitat disturbance and is thus a good candidate for reafforestation of degraded areas^39^. Our hypotheses were that leaves in the dry season would exhibit morphoanatomical characteristics associated with reduced photosynthesis, such as a thinner lamina and smaller midrib xylem in relation to the wet season.

## Results

### Leaf anatomy

**Figure 1:**
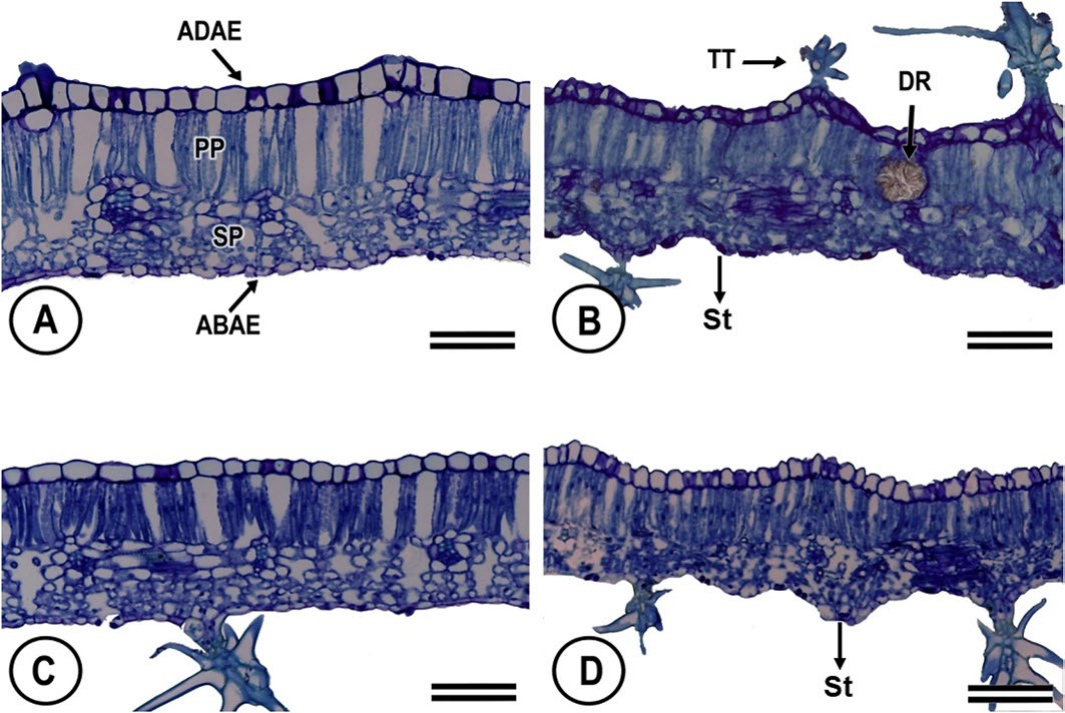
Light micrographs of cross-sections of the leaves in *Croton blanchetianus* plants collected during the wet season (May/July; **A**,**B**) and dry season (October/December, **C**,**D**) of 2015 in full sun (**A**,**C**) and natural shade (**B**,**D**) plants. ADAE, adaxial epidermis surface; PP, palisade mesophyll; SP, spongy mesophyll; ABAE, abaxial epidermis surface; TT, tector trichomes; DR, druse; St, stomatal. Scale = 100 $\upmu$m. Data were collected during wet season (May/July) and dry season (October/December) of 2015.

**Figure 2:**
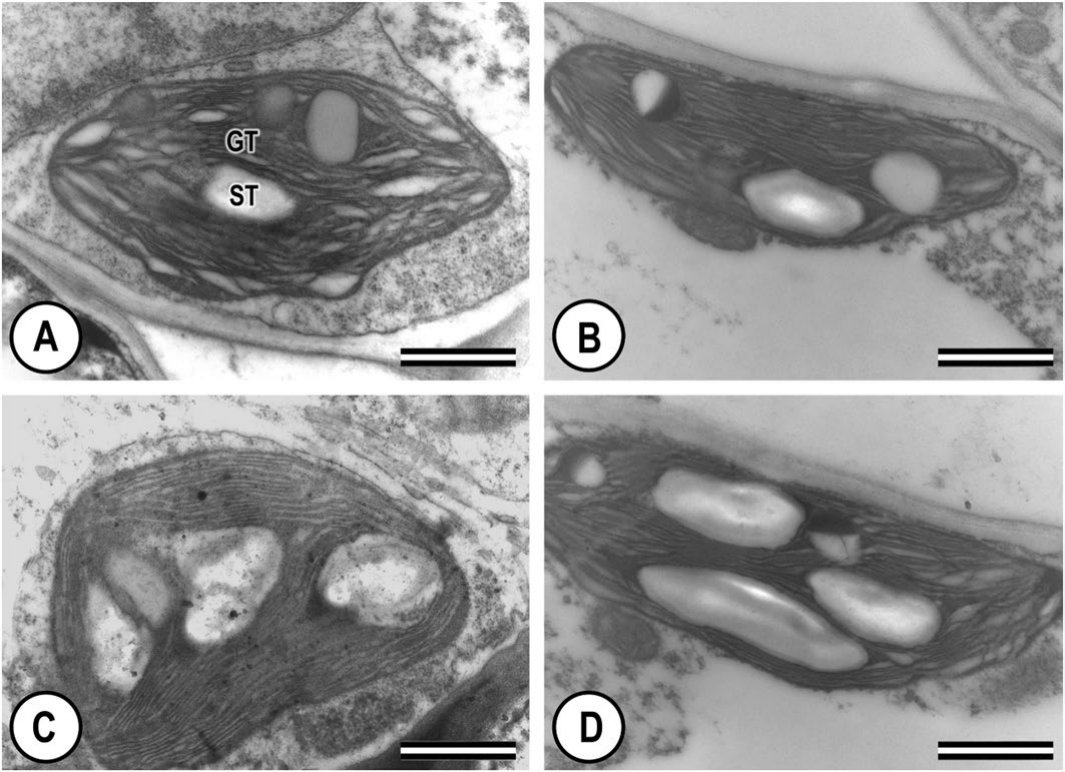
Transmission electron microscopy of the chloroplasts and starch grains in *Croton blanchetianus* plants collected during the wet season (May/July; **A**,**B**) and dry season (October/December, **C**,**D**) of 2015 in full-sun (**A**,**C**) and natural shade (**B**,**D**) plants. GT, grana thylakoids; ST, starch grains. Scale = 1 $\upmu$m.

**Figure 3:**
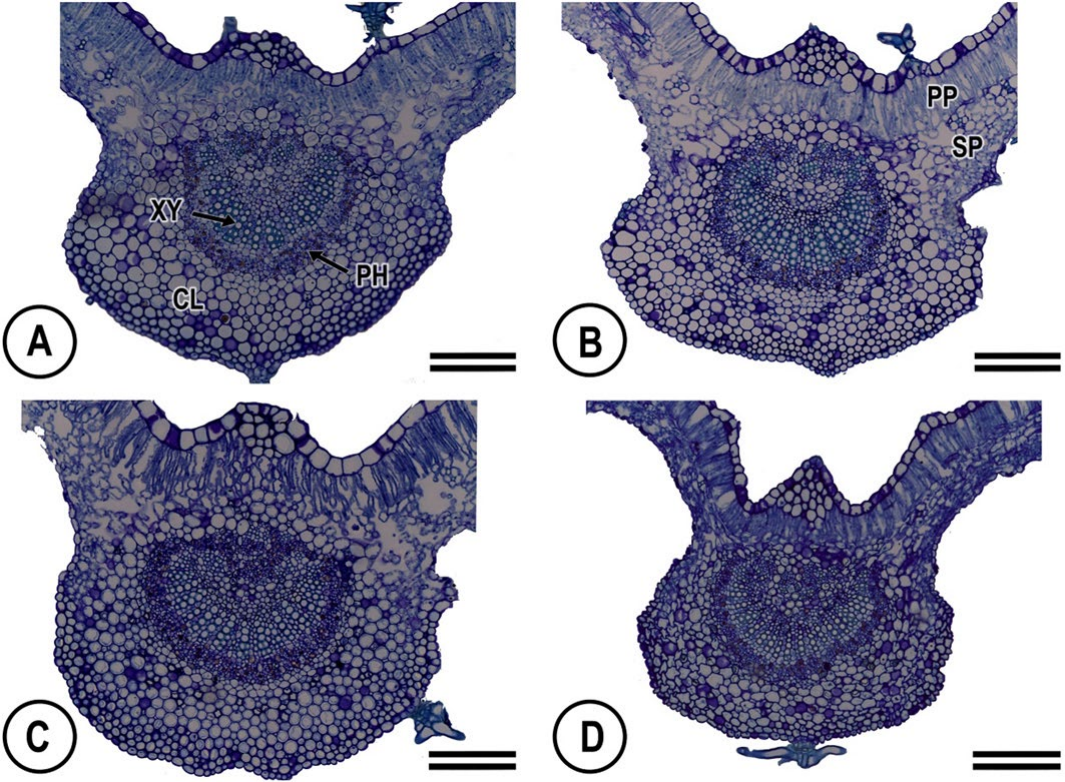
Light micrographs of cross-sections of the midrib in *Croton blanchetianus* plants collected during the wet season (May/July; **A**,**B**) and dry season (October/December, **C**,**D**) of 2015 in full-sun (**A**,**C**) and natural shade (**B**,**D**) plants. XY, xylem; PH, phloem; CL, collenchyma; PP, palisade mesophyll; SP, spongy mesophyll. Scale = 100 $\upmu$m.

Both abaxial and adaxial leaf epidermises of *C. blanchetianus* are uniseriate with anticlinal outer cell walls covered by a thin cuticle, and containing numerous qualitatively observed tector trichomes (Fig. 1). Abaxial epidermal cells are substantially smaller than adaxial. The abaxial surface contains stomata, and stellate and lepidote trichomes. Mesophyll is organized dorsiventrally with a clear distinction between palisade and spongy mesophyll. The palisade mesophyll is a single layer of elongated cells punctuated with an occasional druse of large calcium oxalate crystals. Chloroplasts are ellipsoid with thylakoids organized in grana (stacked) and stroma (unstacked) lamellae (Fig. 2). The midrib is biconvex and it’s epidermis is uniseriate with a thin cuticle, and contains numerous tector trichomes of the star type (Fig. 3). Vascular bundles of the midrib and lateral veins are collateral, with phloem fibers around each bundle (Fig. 3).

### Season and shade

**Figure 4:**
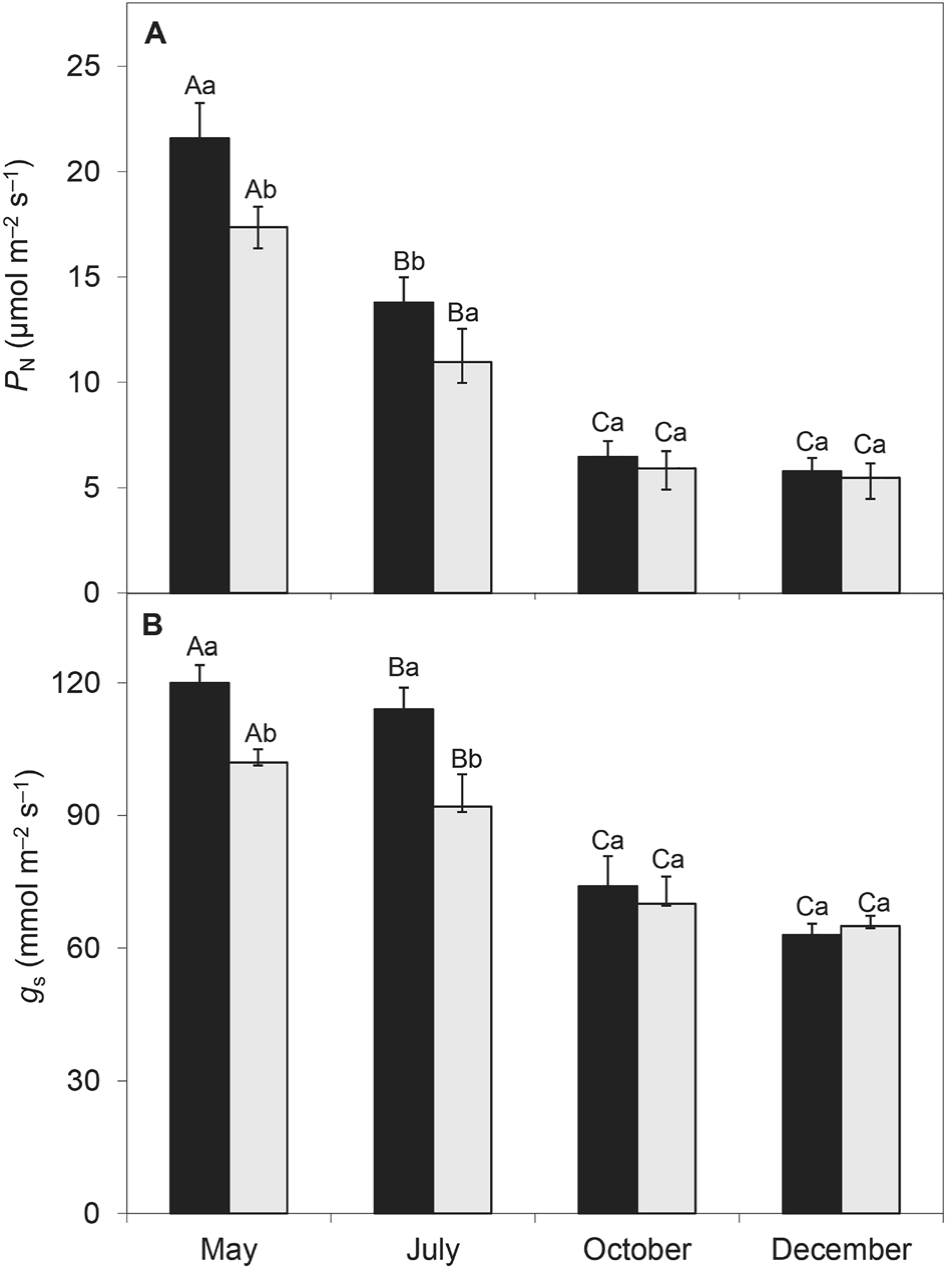
Net photosynthesis per unit mass (*P*_N_, **A**) and stomatal conductance (*g*_s_, **B**) measured for full-sun (black bars) and natural-shade plants (open bars) of *Croton blanchetianus*. Data were collected during the wet season (May/July; **A**,**B**) and dry season (October/December, **C**,**D**) of 2015. Different capital letters denote significant differences among the means for each month within the same light condition, and different lowercase letters denote significant differences between light conditions within the same month. Each value represents the average (± SE), n = 20.

**Figure 5:**
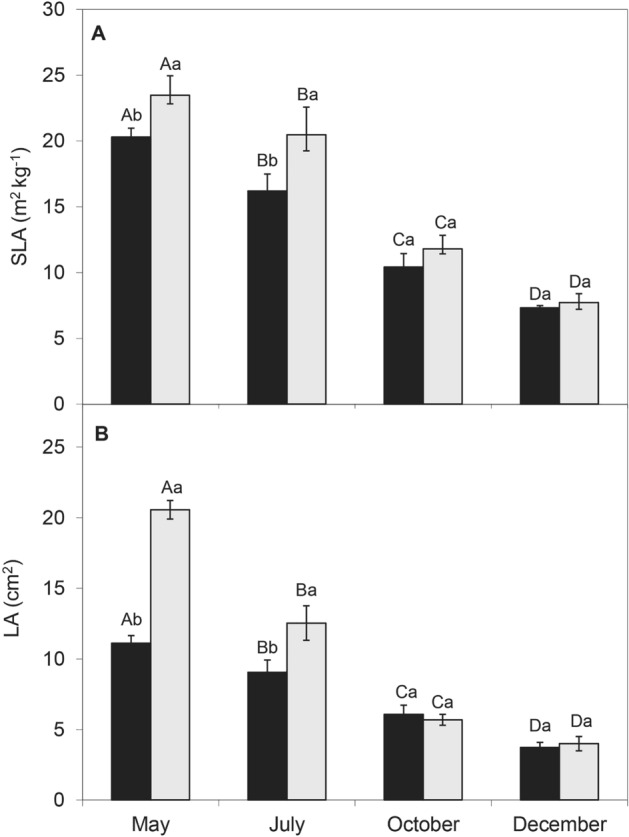
Specific leaf area (SLA, **A**) and leaf area (LA, **B**) measured for full-sun (black bars) and natural-shade plants (open bars) of *Croton blanchetianus*. Data were collected during the wet season (May/July) and dry season (October/December) of 2015. Different capital letters denote significant differences among the means for each month within the same light condition, and different lowercase letters denote significant differences between light conditions within the same month. Each value represents the average (± SE).

All measured leaf parameters were significantly different (*p* < 0.01) between wet and dry seasons. Net photosynthesis was 21.58 µm^−2^ s^−1^ and 17.35 µm^−2^ s^−1^ in the wet season, compared to 5.78 µm^−2^ s^−1^ and 5.47 µm^−2^ s^−1^ in the dry season, for full-sun and shaded treatments respectively, and stomatal conductance was 1.6 and 1.7 times higher (Fig. 4). Shaded leaves in the wet season had higher specific leaf area (19.9 m^2^ kg^−1^ in full-sun and 23.1 m^2^ kg^−1^ in shade), but in the dry season they did not differ from those in full sun (7.5 m^2^ kg^−1^ compared to 7.2 m^2^ kg^−1^, respectively) (Fig. 5).

Table 1 shows that the thickest leaves were those growing under full sunlight during the wet season. Leaves grown in full sunlight were 23% thicker than shaded leaves in both wet and dry seasons, due to an increased thickness of the adaxial epidermis (27%) and both the palisade (21%) and spongy mesophyll (30%, Table 1). There was also a lower percentage of mesophyll airspaces in full sunlight leaves. Abaxial epidermal thickness was unaffected by shading (*p* > 0.05).

**Table 1:** Leaf morphological variation of *Croton blanchetianus*, grown in full sunlight and natural shade. Data were collected during the wet season (May/July) and dry season (October/December) of 2015. Capital letters denote significant differences between months within the same light conditions, and lower-case letters denote significant differences between each light condition within the same month (*p* ≤ 0.001, Newman–Keuls test). The values represent the mean of 20 replicates.

Parameters	Full sunlight				Natural shade			
	May	July	October	December	May	July	October	December
**Lamina thickness**								
Leaf (μm)	236.1 Aa	221.8 Ba	216.4 Ba	197.8 Ca	196.7 Ab	163.5 Bb	162.3 Bb	151.3 Cb
Adaxial epidermis (μm)	30.8 Aa	31.6 Aa	32.2 Aa	31.0 Aa	26.0 Ab	22.5 Bb	20.8 Bb	24.5 Bb
Abaxial epidermis (μm)	11.9 Aa	11.2 Aa	11.3 Aa	11.7 Aa	11.2 Aa	11.1 Aa	11.2 Aa	11.8 Aa
Palisade mesophyll (μm)	106.1 Aa	97.8 Ba	96.4 Ba	82.6 Ca	92.0 Ab	69.1 Bb	70.3 Bb	70.5 Bb
Spongy mesophyll (μm)	88.8 Aa	82.6 Ba	77.5 Ba	73.2 Ca	69.8 Ab	62.6 Bb	50.4 Bb	45.4 Cb
**Lamina air spaces**								
Palisade mesophyll (%)	7.0 Bb	7.2 Bb	7.1 Bb	10.9 Ab	12.6 Aa	12.0 Aa	14.3 Ba	14.6 Ba
Spongy mesophyll (%)	19.5 Cb	20.1 Cb	32.5 Bb	35.9 Ab	29.4 Ba	29.2 Ba	38.2 Aa	38.8 Aa
**Midrib**								
Thickness (mm)	1.004 Aa	0.980 Aa	0.736 Ba	0.688 Ba	0.916 Ab	0.928 Ab	0.764 Ba	0.630 Ca
Length (mm)	1.169 Aa	1.014 Ba	0.823 Ca	0.821 Ca	1.011 Ab	0.968 Ab	0.834 Ba	0.690 Cb
Area (mm^2^)	0.858 Aa	0.843 Aa	0.378 Ba	0.349 Ba	0.622 Ab	0.650 Ab	0.385 Ba	0.257 Cb
**Midrib xylem**								
Thickness (μm)	80.8 Aa	86.3 Aa	79.7 Aa	66.7 Ba	67.6 Ab	70.5 Ab	63.9 Ab	52.4 Bb
Area (mm^2^)	0.082 Aa	0.081 Aa	0.038 Ba	0.027 Ca	0.049 Ab	0.042 Bb	0.031 Cb	0.022 Db
**Vessel**								
Area (μm^2^)	65.5 Aa	59.6 Aa	37.4 Ba	28.7 Ca	69.9 Aa	58.3 Ba	38.5 Ca	30.0 Da
Number of cells	204 Aa	194 Aa	111 Ba	83 Ca	126 Ab	120 Ab	91 Bb	70 Cb

All measured midrib parameters were largest in leaves grown in full sunlight in the wet season (*p* < 0.01). However, shading did not significantly effect the vessel area (*p* > 0.05) (Table 1).

Chloroplast thickness, length and area were significantly larger in the wet season in both full sunlight (2.1 μm, 5.1 μm, 15.2 μm^2^; respectively) and shaded plants (2.0 μm, 5.2 μm, 14.8 μm^2^ ; respectively), and the number of starch grains was larger (4.3 in full sunlight and 4.6 in natural shade) in the dry season when compared to the rainy season (1.5 in full sunlight and 1.7 in natural shade) (Table 2).

**Table 2:** Chloroplast structure in full sunlight and natural shade leaves in field-grown *Croton blanchetianus* plants. Data were collected during the wet season (May/July) and dry season (October/December) of 2015. Capital letters denote significant differences between months within the same light conditions, and lower-case letters denote significant differences between each light condition within the same month (*p* ≤ 0.001, Newman–Keuls test). The values represent the mean of 20 replicates.

Chloroplast parameters	Full sunlight		Natural shade	
	May	December	May	December
Thickness (μm)	2.1 Aa	1.6 Ba	2.0 Aa	1.2 Bb
Length (μm)	5.1 Aa	4.1 Ba	5.2 Aa	3.1 Bb
Area (μm^2^)	15.2 Aa	7.9 Ba	14.8 Aa	5.3 Bb
Starch area (%)	13.7 Ba	30.5 Aa	9.5 Bb	32.8 Aa
Number of starch grains	1.5 Ba	4.3 Aa	1.7 Ba	4.6 Aa

### Multivariate analysis

**Figure 6:**
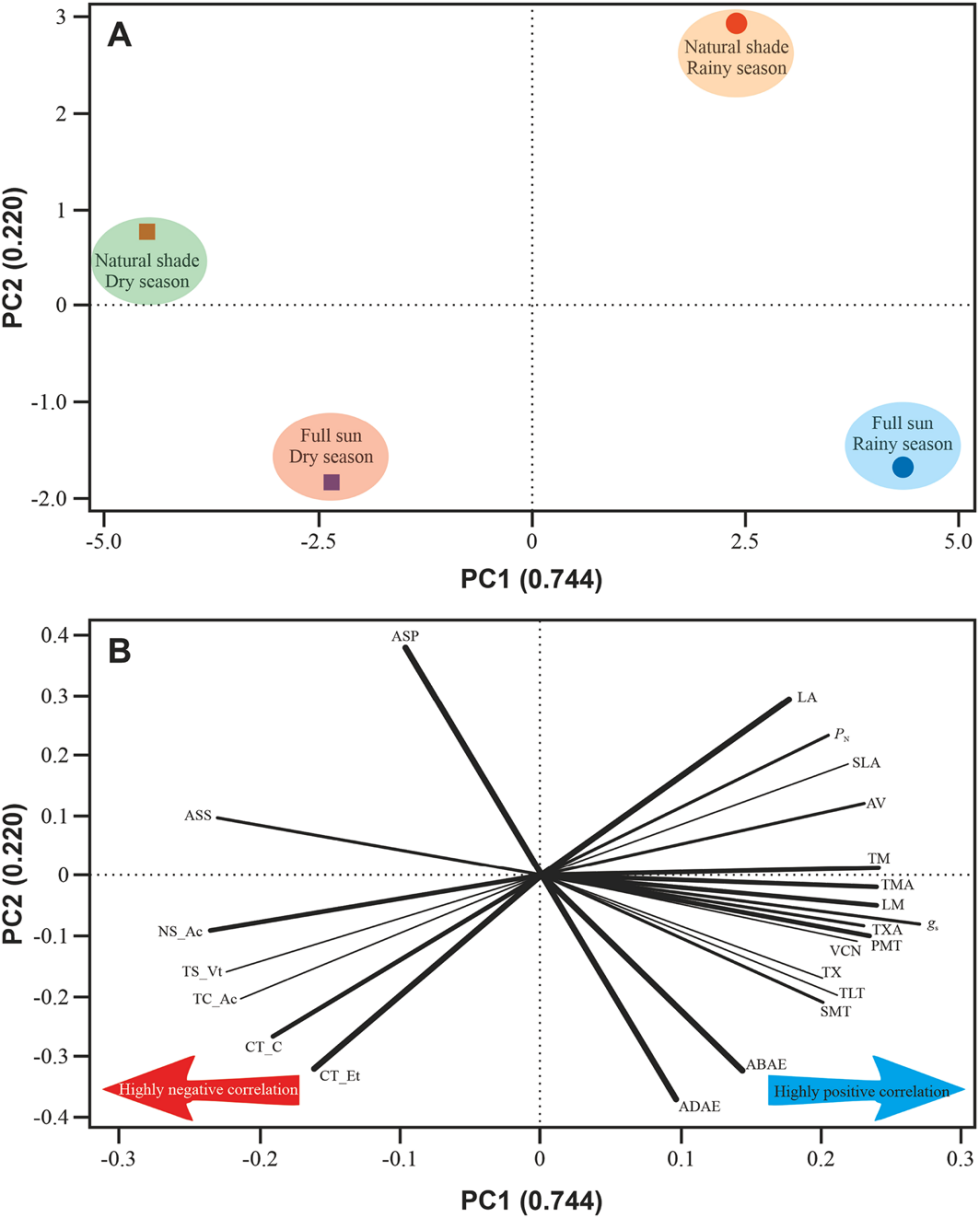
(**A**) Principal component analysis (PCA) of the physiological and anatomical parameters of *Croton blanchetianus*. In A, the large polymons represent the four clusters formed by the Euclidean distance method considering ~ 80% of similarity. (**B**) Loading plot of the physiological and anatomical parameters of *Croton blanchetianus*. In the loading plot the direction. length, and strength (thickness) of the lines are directly proportional to variables importance in separating groups. PC1, principal component 1; PC2, principal component 2. *P*_N_, net photosynthesis per unit mass; *g*_s_, stomatal conductance; SLA, specific leaf area; LA, leaf area; TLT, total leaf thickness; ADAE, adaxial epidermis thicknesses; ABAE, abaxial epidermis thicknesses; PMT, palisade mesophyll thickness, SMT, spongy mesophyll thickness; ASP, air spaces in palisade mesophyll; ASS, air spaces in spongy mesophyll; TM, thickness of the midrib; LM, length of the midrib; TMA, total midrib area; TX, thickness of xylem; TXA, total xylem area; AV, area of vessel; VNC, vessel number of cells; CT_Et, chloroplast thickness; CL_C, chloroplast length; TC_Ac, total chloroplast area; TS_Vt, total starch area; NS_Ac, number of starch grains.

All measured variables were used in the principal components analysis (Fig. 6). Groups were determined by Euclidean distance with similarity of at least 79.6%. Through the principal components analysis results, a clear influence of drought stress modulating some morphoanatomical and ecophysiological attributes could be observed. The PCA showed that treatment groups were 79.6% distinct to each other. Figure 6 shows strong, moderate and weak features that promote separation or clustering of treatment groups. In the upper-right quadrant we verify that LA (29.4%) and TM (24.1%) have a strong influence on separating natural shade in the rainy season from other treatment groups, while natural shade in the dry season were driven mainly by ASP (38%—upper-left quadrant) and weakly, but significantly by ASS (23.1%). On the other hand, full sun in the rainy season (lower-right quadrant) was strongly driven by ADAE (37.4%) and ABAE (32.4%), while full sun in the dry season (lower-left quadrant) was strongly modulated by CT_Et (32.3%) and CT_C (26.7%), and weakly, but very significantly by NS_Ac (23.6%).

## Discussion

This study shows that leaf morphometry and net photosynthesis of *Croton blanchetianus* adjusts seasonally and in response to shade. Water availability and transpiration are the main factors distinguishing treatments, although irradiance also vary. Thus, observed variations were mostly explained by season (wet vs dry) but shading (full sun vs natural shade) was also significant. To our knowledge, this study is the first to combine leaf morphometrics and chloroplast ultrastructure with gas exchange in *Croton blanchetianus.*

Leaf water deficit is the primary limiting factor for net photosynthesis, and sub-optimal metabolic conditions during severe stress can further reduce it^40,41^. Our results corroborate previous findings of seasonal photosynthetic variation in Caatinga species^4^ and other species^42^. Mendes et al^4^ observed *P*_N_ between 15–20 µmol m^−2^ s^−1^ in the rainy season and less than 5 µmol m^−2^ s^−1^ in the dry season in a study with the species *Croton blanchetianus*. Pinho-Pessoa et al^43^ found a *P*_N_ variation from 13.3 µmol m^−2^ s^−1^ in the rainy season to 6.7 µmol m^−2^ s^−1^ in *Cenostigma pyramidale*, considered an endemic species in the Caatinga.

Leaf area and thickness declined significantly in the dry season in newly produced leaves. This volumetric reduction under periods of water deficit is associated with reduced cell expansion in the palisade mesophyll. Water-deficit stress can reduce leaf growth and, in turn, leaf area, because leaf area expansion depends on leaf turgor, temperature, and assimilating supply for growth, which can be affected by drought as observed in several species^4,44–47^.

In this study, we showed a larger leaf area in full-sun leaves developed in the rainy season. Since there is a cost to the expression of morphological traits, it is likely that plants exhibit optimal expression, *i.e.,* greater specific and absolute leaf area during the wet season, when greatest photosynthetic activity is observed. Gotsch^48^ and Mendes^4^ found that specific leaf area is generally higher in the dry forest, particularly during the wet season. It is important to emphasize that there was no significant difference in absolute or specific leaf area between leaves in full-sun and shade in the dry season, probably because the light penetration in dry forests is higher during the dry season when many canopy and understory species shed their leaves, which is no usually observed in rainforests. Our results are in accordance with other studies^47,49,50^, that investigated leaf acclimation (absolute and specific leaf area) to irradiance. Leaves suffer a negative pressure in relation to the atmosphere, which usually presents a negative water potential of up to −100 MPa. Thus, leaf is pressured to lose water to the atmosphere by transpiration. However, since it can die from lack of water, its stomata close and nearly ends transpiration or latent heat fluxes. Thus, non-transpiring closed-stomata leaves increase their temperature, which is also negatively correlated with photosynthesis. On the other hand, native plants from dry and xerophytic environments develop signs that lead to the development of smaller and thicker leaves^51^. Large leaves have smaller convection coefficients and a higher resistance to heat transfer through leaf boundary layers than smaller leaves, and leaf size may change in order to optimize leaf temperature^52^. Larger leaves may be intrinsically vulnerable to drought-induced embolism due to the lower vein length and larger xylem conduit diameters^53^.

The arrangement of a thin cuticle in *C*. *blanchetianus* indicates that not always an endemic species of a region subjected to high temperatures, high irradiance, and low rainfall indexes must necessarily deposit a thick epidermal hydrophobic barrier. Chazdon and Kaufmann^54^ argue that this structure plays an important role in increasing light reflection, which would help the leaf to protect itself against excessive irradiation. However, in *C*. *blanchetianus* it is possible that only a portion of this function is fulfilled by the cuticle. In fact, this activity is more effectively fulfilled by a large number of tector trichomes present along the entire leaf surface, which would act as a protective barrier against the damaging action of ultraviolet rays on structures that participate in the process of photosynthesis^55,56^.

The occurrence of stomata only on the abaxial surface of the epidermis may be useful in controlling water loss to the environment. The fact that in *C*. *blanchetianus* the stomata are arranged at upper portions of the epidermis, which also differs from the xeromorphic pattern, in which stomata are located in depressions, would be favored by the existence of a microenvironment formed by intense coverage of trichomes on these regions, controlling the loss of water to the surrounding air^57,58^. As emphasized by Glover^47^, trichomes are prominent in saving water. Specifically, the non-glandular trichomes, such as those described here in *C. blanchetianus*, have been extensively described^57–59^ as hairs providing shade on the leaf surface to maintain a humid layer and reduce water loss through evaporation especially when stomata are open.

The arrangement of a palisade mesophyll composed of a single cell layer is quoted for the genus *Croton*, so that such arrangement may be directly associated with the channeling of the light path within the leaf^60^. For the sake of clarity, in this paper we describe that full-sun plants had leaves with greater total thickness while natural shade plants have a smaller palisade and spongy mesophyll compared to the full-sun leaves. It is important to note that in the wet season both full-sun and natural shade leaves were thicker; the opposite was observed in the dry season showing that the seasonality of precipitation can influence the mesophyll thickness (Table 1, Fig. 1). Several studies showed the relationship between leaf thickness and light intensity^61–65^. The increase of leaf thickness with the increase of irradiance is a mechanism that may increase photosynthesis per unit leaf area and enable better water-use efficiency^33,66^.

Principal component analysis (Fig. 6) showed the distinct functional groups that stand out for their water economy in the driest periods and the plasticity of this native Caatinga species. The strong correlation between groups shown in Fig. 6 reinforces previous results presented by different authors^3,4,10,13^ that show that native plants from dry environments are very well adapted. The main data reiterate the results of gas, biochemical, enzymatic and nutritional exchanges. Furthermore, correlations between these factors indicate that due to *C. blanchetianus* resilience, it can be considered a model plant for drought resistance studies in open environments in the Caatinga, exceptionally during the driest months.

Transmission electron microscopy revealed that chloroplasts were significantly longer and significantly thicker in the wet season when compared to the dry season, independently of the lightning characteristics. Although we did not analyze a possible correlation between the number of chloroplasts and photosynthetic capacity, transmission electron and light microscopy analyses allow us to argue that chloroplasts were more abundant in mesophyll cells during the wet season when compared to the dry season. In addition, connections among anatomical characteristics of the leaf and midrib can explain the drastic reduction of photosynthesis during droughts. Water deficit may inevitably affect or damage the ultrastructure of chloroplasts. Figure 2 shows that the ultrastructure of chloroplasts during the dry season is more enriched in stroma lamellae than during the wet season. Many studies^12,67,68^ previously described that grana are reduced in stressed chloroplasts and plastoglobuli are more frequent than in non-stressed chloroplasts.

As previously reported in the literature^69^, full-sun leaves showed a higher thickness of xylem and total xylem area compared to shade leaves to compensate for the higher transpiration with an increased water supply (Table 1). Thus, full-sun leaves increase the vessel number, rather than vessel area. On the other hand, in the dry season, leaves presented a lower thickness of xylem, total xylem area, vessel area and number compared to the wet season. As shown in other studies^38,70,71^ these features indicate that, in these plants, water availability is more likely to modulate the hydraulic system of water transport.

Interestingly, all the characteristics of the midrib were positively correlated with net photosynthesis. Our study suggests that changes in the characteristics of the vein xylem may support hydraulic control for gas exchange during adaptation to contrasting light regimes as well as water deficit. This would suggest that the studied species modulate their midrib characteristics, such as the reduction area of vessel and density of vessel as a strategy for their tolerance to the lower availability of water in the dry season. It is known that lesser area of vessel prevents embolism which results in cavitation and may limit plant productivity^35,72^. These changes in vein xylem conductivities can drive impacts on leaf gas exchange and its responses to external conditions. This statement is supported by published correlation analysis between leaf hydraulic conductance and leaf anatomy^73^. Scoffoni^73^ studied the relationship between leaf hydraulic conductance and leaf anatomy for six ecologically diverse species at high and low irradiance. These researchers observed that across species, anatomical changes statistically explained 40% of the observed variation in leaf hydraulic conductance. McKown et al^36^ reported that plants grown under higher irradiance tended to develop smaller, thicker leaves and with higher major vein length per area, which are traits that would increase vein xylem conductivities. Our results show high variation in the midrib traits, mainly in total midrib area, total xylem area and area of vessel. This is in agreement with other studies^37,71^.

We have proved that the magnitude of the seasonal variation of rainfall is responsible for altering the correlations among morphoanatomical traits and photosynthesis mainly during the dry season. In agreement with our results, the correlations between absolute and specific leaf area, total leaf thickness, palisade and spongy mesophyll thickness, air spaces in the palisade mesophyll, xylem thickness, total xylem area, vessel number and net photosynthesis were stronger in the dry season. Therefore, it is possible to identify a close relationship between absolute and specific leaf area, total leaf thickness, and net photosynthesis in *C. blanchetianus*. Also, our research is consistent with previous studies^4,9,74,75^ which showed that morphophysiological traits can be responsive to rainfall-related environmental factors. Lastly, our results indicate that *C*. *blanchetianus* may adapt to a new, drier environment and/or under greater light availability by altering patterns of leaf-trait correlations (*i.e.,* phenotypic integration).

## Summary and conclusions

In summary, our results have presented morphoanatomical and ecophysiological mechanism responsible for drought stress tolerance in *C*. *blanchetianus*, based on seasonal data. Leaf traits of *C*. *blanchetianus* showed structural adaptive mechanisms that are mainly related to water saving. However, the presence of a thin cuticle coating the leaves of this species probably indicates that it will prevent desiccation by using other more effective means, such as structural characteristics of the leaf surface. This fact would justify the wide coverage of tector trichomes along its entire leaf surface. Moreover, our results demonstrate that some leaf traits of the *C*. *blanchetianus* show a high acclimation, particularly some anatomical traits associated with: (i) maintenance of a positive carbon balance in water deficit conditions, and (ii) midrib traits associated with lower leaf hydraulic conductance and also with greater drought tolerance. We demonstrate that physiological characteristics, such as net photosynthesis, can be more sensitive to water deficit, but that leaf anatomical changes may be an adaptive advantage in coping with extended drought stress.

## Material and methods

### Site and species description

Ten 1–3 m tall *C*. *blanchetianus* plants in full sunlight and ten in natural shade were selected from a preserved fragment of Caatinga forest (8° 52′ 32" S, 36° 22′ 00" W; 716 m asl) in the state of Pernambuco, NE Brazil in 2015. *C*. *blanchetianus* is a shrub that can be classified as a semideciduous plant because it loses at least 50% of its leaves every year, never becoming completely deciduous while having a dense crown in the wet season^39^. The Caatinga is a seasonally dry tropical forest under a climate with a Köppen classification of BSh, annual rainfall of 598 mm, and distinct wet (April to September) and dry (October to March) seasons. Average air temperature/humidity during the study period were 20 °C/57% in July (wet season) and 26 °C/77% in December (dry season).

Photosynthetically active light radiation measurement was performed with digital and portable light radiation sensors (Li-1400, Li-Cor, NE, USA) coupled to quantum sensors (Li-190 SA, Li-Cor, NE, USA). Definition of light penetration of only 30%, i.e., 70% of leaf cover, was based on May 2014 conditions, since between April and July 2014 an average precipitation of 148 mm was recorded, with a peak in May 2014 (171.59 mm). Naturally, we could not always guarantee this 70% of vegetation cover, but light penetration inside the forest in the wet season was much lower than that observed at the edges of the forest fragment where the leaves grown in full sunlight were sampled. In turn, leaves grown in natural shade were sampled inside the forest.

Field gas exchange measurements and anatomical analyzes were carried out in May, July, October, and December 2015. After each measurement, leaves at the primordium stage (less than 2 cm in length) were marked with adhesive tape to ensure that following measurements would be conducted on leaves that certainly experienced the conditions portrayed by each of the samplings. Based on the prior knowledge that *Croton blanchetianus* loses approximately 40 to 60% of its leaves in the dry season, we marked between five to 10 leaves to ensure that we would have at least two of these 10 leaves still on the plant during the following measurement. Thus, it can be guaranteed that leaf sampled in May developed between December 2014 and May 2015. This procedure was carried out for all other samplings.

Plants material was sampled in accordance with the Brazilian environmental protection legislation (Law nº 6.938/1981). Data measurement was authorized by the Chico Mendes Institute for Biodiversity Conservation (ICMBio) under sampling permit SISBIO 28,310, issued by the Biodiversity Authorization and Information System (SISBIO, IN ICMBio nº 03/2014).

### Gas exchange

Net photosynthesis (*P*_N_) and stomatal conductance (g_s_) were measured on two fully expanded leaves from each of the 20 shrub in May, July (wet season), October and December 2015 (dry season) using a portable infrared gas analyzer system (Li-6400; Li-Cor, NE, USA). Individual leaves were exposed to saturating irradiance of 1000 µmol m^−2^ s^−1^, ambient CO_2_ concentration (390 ppm), and airflow of 400 μmol s^−1^. Measurements were conducted in situ in full sunlight (non-cloudy conditions) for two hours starting 8 am. This time of day was selected after preliminary experiments indicated that stomatal conductance would be greatest at this time.

### Leaf morphometrics

The same leaves in which gas exchanges were measured were sampled for the anatomical and ultrastructural studies. So, leaf containing midrib and lamina were obtained from the lengthwise midpoint of the leaf. Two samples were obtained from each of four leaves, taken from the 20 plants (10 in full sun, 10 shaded) in May, July (wet season), October and December (dry season) 2015. Leaf samples, 160 blade fragments, were fixed in FAA_50%_ for 48 h and then stored in 70% (v/v) ethanol until analysis.

Leaf samples were dehydrated in an ethylic series and embedded in plastic resin (Historesin-Leica Microsystems Nussloch, Heidelberg, Germany). Paradermic layers (4 μm) and cross-sections (7 μm) were obtained with a rotary microtome (Model RM2255, Leica Microsystems Inc., Deerfield, USA), stained with toluidine blue at pH 4.0^76^ and mounted in synthetic resin (Fisher Chemical™ Permount™ Mounting Medium, part number SP15-500, Sunnyvale, CA USA). Images were captured with a light microscope (Leica Mikrosysteme Vertrieb GmbH, model DM750; Wetzlar, Germany) equipped with a digital camera (Mikrosysteme Vertrieb GmbH, model ICC50 HD; Wetzlar, Germany) interfaced to a computer.

Morphometric parameters (see Table 1) were measured using Image Pro® Plus, (version 4.5.0.29, Media Cybernetics, Silver Spring, USA). The following anatomical data were measured: (i) total leaf thickness; (ii) adaxial and abaxial epidermal thicknesses; (iii) palisade and spongy mesophyll thicknesses; (iv) palisade and spongy air space, computed as a percentage of the total cross-sectional area of the mesophyll tissue; (v) thickness, length and area of the midrib; (vi) thickness and area of the midrib xylem and (vii) vessel area and number of vessel cells. Measurements were randomly conducted using 20 light micrographs (10 times per micrograph) per plant on each season, for: total leaf thickness, adaxial and abaxial epidermal thicknesses, palisade and spongy mesophyll thicknesses, thickness and length of the midrib, thickness and length of the midrib xylem. Epidermal thicknesses was measured considering the distance between the inner and outter sides of the periclinal wall of each epidermal cell, that is, the cross-sectioned length of the cells. The vessel area was measured considering all the largest vessels located in the vascular bundle, thus standardizing leaf comparison. In the midrib, number of cells of the vessel was calculated considering all visible vessels in the vascular bundle.

### Chloroplast morphometrics

Samples for leaf morphometrics were also used for chloroplast morphometrics. Some 5 mm fragmens were sampled from each plant, and were fixed in Karnovsky buffer^77^ for 30 days, then washed three times in cacodylate buffer (sodium cacodylate trihydrate, part number C0250, Sigma Aldrich, St. Louis, USA) and post-fixed with 1% (v/v) osmium tetroxide (part number 201030, Sigma Aldrich, St. Louis, USA) in 0.05 M phosphate buffer, dehydrated in a series of increasing acetone concentration, infiltrated and polymerized in low viscosity epoxy resin^78^. Cross sections of 70 nm thickness were obtained using an ultramicrotome (Power Tome-X, RMC Products, Boeckeler Instruments, Inc., Tucson, USA) and stained with uranyl acetate, then lead citrate^79^. Chloroplast parameters (see Table 2) were obtained with a transmission electron microscope, 50 kV, with a coupled digital camera (EM 109, Carl Zeiss Microscopy Ltd., Jena, Germany).

### Total leaf area and specific leaf area

To determine the phenology of the leaves during the study period, we carefully sampled only leaves that had completed their development in each season. Leaf area was measured for 200 healthy and expanded leaves collected in each season from both full sun and natural shade plants.The leaves were randomly sampled from different parts of 20 different shrub. All leaves were digitalized and the images were analyzed using Image® Pro Plus software. The specific leaf area (SLA) was determined using leaves similar to those used in the gas exchange measurements. Leaf dry mass was obtained after oven-drying at 70 °C until a constant mass was achieved and weighed when the SLA (m^2^ kg^−1^) was estimated.

### Univariate analysis

Effects of sun exposure (full sun, natural shade), and either season (wet, dry) or month (May, July, October, December), were assessed using a repeated measures ANOVA and means were compared through Tukey’s test. The experimental design contained multiple nested layers; e.g.; leaf morphometrics were measured on 10 random points nested within 20 photomicrographs, each nested within two leaf samples, each nested within four leaves, each nested within 10 plants, nested within two sunlight regimes. However, nesting below the level of plant was ignored in the statistical model after preliminary analysis showed data within plants conformed to both normality (Shapiro–Wilk test) and homoscedasticity (Brown-Forsyth test). Analysis was conducted using Statistica version 10.0 (StatSoft, Tulsa, OK, USA).

### Multivariate analysis

A canonical correlation analysis was conducted to explore the influence of leaf morphometrics on net photosynthesis^80^. Pearson’s correlation coefficient was used to examine the relationships among measured variables. Rand Index was used to measure the similarity between data groupings and the Euclidian distance was used for principal components analysis using Minitab 18.1 (Minitab LLC, Pennsylvania State University, PA, USA). Results were considered significant when *p* ≤ 0.01. Principal component analysis was conducted on physiological and morphological parameters using Minitab 18.1.0.0 (Minitab LLC, State College, PA, USA). The summary function of principal components analysis was used to calculate the proportion of the variance of each parameter explained by each principal component. For hierarchical clustering, Pearson’s correlations were used to compare the similarities between the studied species using the “cor” function of the R stats package, and the complete linkage method and the Euclidean distance measure were used for hierarchical clustering with the R index in the Minitab 18.1 (Minitab LLC, Pennsylvania State University, PA, USA).

## Supplementary Information

Supplementary Information.
