# Supplementary material for: Leaf plasticity across wet and dry seasons in Croton blanchetianus (Euphorbiaceae) at a tropical dry forest
Source: Sci Rep. 2022 Jan 19;12:954. doi: 10.1038/s41598-022-04958-w (PMC8770520; doi:10.1038/s41598-022-04958-w)
Supplement: Supplementary file 1 — Supplementary Information. [file 41598_2022_4958_MOESM1_ESM.docx]

**Supplementary Table S1.** Over-representation analysis of the principal component (PC) variable loadings the physiological, anatomical and ultrastructural with a main impact on the photosynthesis variance. All parameters contributions in each PC was measured in *Croton blanchetianus* analysed in full sun and natural shade, both in rainy season and dry season. The plants were exposed to natural ambient without treatments, where the objectives were analyse de shade and light on photosynthesis. *P*_N_, net photosynthesis per unit mass; *g*_s_, stomatal conductance; SLA, specific leaf area; LA, leaf area; TLT, total leaf thickness; ADAE, adaxial epidermis thicknesses; ABAE, abaxial epidermis thicknesses; PMT, palisade mesophyll thickness, SMT, spongy mesophyll thickness; ASP, air spaces in palisade mesophyll; ASS, air spaces in spongy mesophyll; TM, thickness of the midrib; LM, length of the midrib; TMA, total midrib area; TX, thickness of xylem; TXA, total xylem area; AV, area of vessel; VNC, vessel number of cells; CT_Et, chloroplast thickness; CL_C, chloroplast length; TC_Ac, total chloroplast area; TS_Vt, total starch area; NS_Ac, number of starch grains.

| **Variable** | **CP1** | **CP2** | **CP3** | **CP4** | **CP5** | **CP6** | **CP7** | **CP8** | **CP9** | **CP10** |
| --- | --- | --- | --- | --- | --- | --- | --- | --- | --- | --- |
| *P*_N_ | 0.205 | 0.233 | 0.099 | 0.207 | -0.202 | -0.055 | -0.279 | 0.174 | 0.165 | 0.294 |
| *g*_s_ | 0.234 | -0.072 | -0.21 | 0.18 | -0.47 | -0.04 | 0.264 | -0.295 | 0.059 | -0.298 |
| SLA | 0.219 | 0.186 | -0.02 | -0.143 | -0.033 | -0.204 | -0.002 | -0.312 | -0.207 | 0.35 |
| LA | 0.177 | 0.294 | 0.182 | 0.135 | -0.019 | 0.009 | 0.166 | -0.111 | 0.268 | -0.198 |
| TLT | 0.212 | -0.199 | 0.194 | -0.038 | 0.134 | -0.033 | 0.207 | -0.117 | 0.018 | 0.34 |
| ADAE | 0.096 | -0.374 | 0.409 | 0.059 | -0.186 | 0.004 | 0.101 | -0.028 | 0.012 | 0.143 |
| ABAE | 0.144 | -0.324 | 0.375 | 0.069 | 0.228 | 0.466 | -0.24 | -0.263 | -0.103 | -0.145 |
| PMT | 0.235 | -0.102 | 0.017 | 0.033 | 0.02 | 0.076 | 0.254 | 0.176 | 0.294 | -0.115 |
| SMT | 0.202 | -0.213 | 0.298 | -0.175 | -0.212 | -0.048 | -0.013 | 0.166 | 0.137 | -0.152 |
| ASP | -0.097 | 0.38 | 0.363 | -0.03 | -0.348 | 0.233 | 0.06 | -0.014 | -0.515 | 0.038 |
| ASS | -0.231 | 0.096 | 0.217 | 0.047 | 0.12 | -0.221 | -0.038 | 0.178 | -0.107 | -0.369 |
| TM | 0.241 | 0.014 | -0.034 | -0.134 | 0.093 | -0.127 | -0.191 | 0.182 | -0.27 | -0.419 |
| LM | 0.24 | -0.052 | -0.016 | -0.008 | -0.129 | -0.347 | -0.101 | -0.096 | 0.107 | -0.105 |
| TMA | 0.238 | -0.044 | -0.152 | 0.059 | 0.053 | 0.293 | 0.449 | 0.535 | -0.284 | 0.069 |
| TX | 0.217 | -0.186 | 0.151 | 0.169 | 0.066 | -0.506 | -0.186 | 0.191 | -0.329 | 0.085 |
| TXA | 0.228 | -0.092 | -0.294 | 0.017 | -0.065 | 0.139 | -0.082 | -0.365 | -0.349 | -0.183 |
| AV | 0.231 | 0.121 | -0.137 | 0.027 | -0.144 | 0.294 | -0.429 | 0.136 | 0.144 | 0.206 |
| VNC | 0.225 | -0.109 | -0.296 | 0.005 | 0.118 | 0.038 | -0.079 | 0.116 | -0.133 | 0.065 |
| CT_Et | -0.162 | -0.323 | -0.174 | -0.178 | -0.123 | 0.12 | -0.327 | 0.108 | 0.063 | -0.026 |
| CL_C | -0.191 | -0.267 | -0.14 | -0.144 | 0.006 | -0.155 | 0.234 | -0.106 | -0.119 | 0.225 |
| TC_Ac | -0.214 | -0.204 | -0.042 | -0.111 | -0.603 | -0.01 | -0.044 | 0.19 | -0.058 | 0.015 |
| TS_Vt | -0.225 | -0.161 | -0.071 | 0.841 | -0.02 | -0.004 | -0.045 | -0.011 | -0.086 | 0.037 |
| NS_Ac | -0.236 | -0.091 | 0.048 | -0.137 | -0.004 | 0.000 | 0.006 | -0.099 | 0.004 | 0.000 |
